# Diabetes prevalence and diagnosis in US states: analysis of health surveys

**DOI:** 10.1186/1478-7954-7-16

**Published:** 2009-09-25

**Authors:** Goodarz Danaei, Ari B Friedman, Shefali Oza, Christopher JL Murray, Majid Ezzati

**Affiliations:** 1 Harvard School of Public Health, Boston, Massachusetts, USA; 2 Initiative for Global Health, Harvard University, Cambridge, Massachusetts, USA; 3 Institute for Health Metrics and Evaluation, University of Washington, Seattle, Washington, USA

## Abstract

**Background:**

Current US surveillance data provide estimates of diabetes using laboratory tests at the national level as well as self-reported data at the state level. Self-reported diabetes prevalence may be biased because respondents may not be aware of their risk status. Our objective was to estimate the prevalence of diagnosed and undiagnosed diabetes by state.

**Methods:**

We estimated undiagnosed diabetes prevalence as a function of a set of health system and sociodemographic variables using a logistic regression in the National Health and Nutrition Examination Survey (2003-2006). We applied this relationship to identical variables from the Behavioral Risk Factor Surveillance System (2003-2007) to estimate state-level prevalence of undiagnosed diabetes by age group and sex. We assumed that those who report being diagnosed with diabetes in both surveys are truly diabetic.

**Results:**

The prevalence of diabetes in the U.S. was 13.7% among men and 11.7% among women ≥ 30 years. Age-standardized diabetes prevalence was highest in Mississippi, West Virginia, Louisiana, Texas, South Carolina, Alabama, and Georgia (15.8 to 16.6% for men and 12.4 to 14.8% for women). Vermont, Minnesota, Montana, and Colorado had the lowest prevalence (11.0 to 12.2% for men and 7.3 to 8.4% for women). Men in all states had higher diabetes prevalence than women. The absolute prevalence of undiagnosed diabetes, as a percent of total population, was highest in New Mexico, Texas, Florida, and California (3.5 to 3.7 percentage points) and lowest in Montana, Oklahoma, Oregon, Alaska, Vermont, Utah, Washington, and Hawaii (2.1 to 3 percentage points). Among those with no established diabetes diagnosis, being obese, being Hispanic, not having insurance and being ≥ 60 years old were significantly associated with a higher risk of having undiagnosed diabetes.

**Conclusion:**

Diabetes prevalence is highest in the Southern and Appalachian states and lowest in the Midwest and the Northeast. Better diabetes diagnosis is needed in a number of states.

## Background

Diabetes Mellitus is the sixth leading cause of death in the United States (U.S.), accounting for approximately 70,000 annual deaths. Age-standardized adult diabetes death rates across U.S. states ranged from approximately 2 per 10,000 people in Arizona and Florida to 4.5 to 5 in West Virginia and the District of Columbia (D.C.) [1]. There may be two reasons for this large variation: First, there may be variation in diabetes prevalence across states due to differences in risk factors for diabetes. For example, the prevalence of obesity in a number of Southern states is almost 60% higher than Colorado, where obesity is lowest [2]. Second, there may be differences across states in diagnosis and treatment of diabetes or of cardiovascular risks among diabetics. Reliable information on diagnosed and undiagnosed diabetes prevalence at the state level is important because states are important administrative units for funding and implementing programs that influence diagnosis and treatment.

Currently, the only source of information on diabetes prevalence at the state level is the Behavioral Risk Factor Surveillance System (BRFSS), a state-representative telephone survey. However, the BRFSS data are based on self-reports and do not provide estimates of undiagnosed diabetes. The National Health and Nutrition Examination Survey (NHANES) uses laboratory measurements and provides estimates of diagnosed and undiagnosed diabetes, but is representative only at the national level. In this study, we combined data from NHANES and BRFSS to estimate diabetes prevalence and diagnosis at the state level. Our results provide information for state diabetes prevention and control programs, and our methods can be used for regular low-cost monitoring of diabetes at the state level.

## Methods

### Data Sources

NHANES uses a complex multistage stratified clustered probability design to measure health and nutrition characteristics of a nationally representative sample of the civilian non-institutionalized population aged two months and older. NHANES includes an in-person interview and a subsequent physical examination and measurement component in a mobile examination clinic (MEC) or at home for those unable to visit the MEC. We used NHANES data from 2003 to 2006. The response rates for the household interviews were 80% for 2003-2004 and 79% for 2005-2006. The corresponding response rates for the medical examination after the household interview were 95 to 96%.

Each interviewed participant was randomly assigned to either a morning or afternoon/evening MEC session. Subjects ≥ 20 years old assigned to the morning session were asked to fast for 8 to 24 hours, with the exception of those on insulin or those who were excluded for other safety reasons. The NHANES MEC and fasting sample weights account for exclusion, non-response, and inappropriate fasting time. Additional information on NHANES design and methods, including on diabetes measurement, is available elsewhere [3,4] and online http://www.cdc.gov/nchs/nhanes.htm.

The BRFSS is an annual cross-sectional telephone health survey. Currently, the survey is conducted in all 50 states and the District of Columbia using random-digit dialing to obtain a state-representative sample of the civilian, non-institutionalized population aged 18 and over. In 2003, the response rate among eligible subjects who answered the phone was 77%. Additional information on the design is available elsewhere [5,6] and online http://www.cdc.gov/brfss.

We included adults aged 30 and older in NHANES and BRFSS who had answered the self-reported diabetes question, which asked if they had ever been told by a health professional that they had diabetes. The response rate for this question was more than 99.8% in both surveys. We did not include younger participants because diabetes prevalence is relatively low in these ages.

### Statistical Analysis

Consistent with previous analyses [4], we defined total diabetes as either having answered yes to the diabetes diagnosis question: "Other than during pregnancy, have you ever been told by a doctor or health professional that you have diabetes or sugar diabetes?" or having a fasting plasma glucose (FPG) level of ≥ 126 mg/dL. We used FPG because it is used to define diabetes by the American Diabetes Association [7].

We used data from NHANES, which is representative at the national but not at the state level, to characterize the relationship between undiagnosed diabetes status (defined as FPG ≥ 126 mg/dL) and a set of health system, sociodemographic, and risk factor variables listed in Table 1 using a logistic regression. These variables were selected a priori based on their potential association with diabetes prevalence. We excluded education from the primary list of predictors as including it did not improve the fit of the model. In addition, 50.2% of observations in NHANES were missing either smoking or insurance status or both. We used a missing indicator to include these observations in the regression model. The regression incorporated appropriate sampling weights.

**Table 1:** Description of the outcome and explanatory variables from NHANES and BRFSS and the corresponding odds ratios (OR) and 95% confidence intervals (95% CI).

Variable	Reason for inclusion in analysis	Possible values	OR	(95% CI)
*Outcome (dependent) variables for the regression*				

Undiagnosed diabetes (available in NHANES; predicted in BRFSS)	Outcome variable to estimate undiagnosed diabetes	0 (FPG < 126 mg/dL) 1 (FPG ≥ 126 mg/dL)	-	-

*Explanatory (independent) variables*				

Sex	Predictor of diabetes, possibly because of differences in lifestyle and health care determinants	Male	1.0	-
	
		Female	0.47	0.29, 0.77

Age (years)	Predictor of diabetes	30-39	1.0	-
		
		40-49	1.07	0.41, 2.81
		
		50-59	3.25	1.41, 7.50
		
		60-69	7.49	3.55, 15.82
		
		≥ 70	7.02	3.23, 15.26

Race *	Predictor of diabetes and health care access	Non-Hispanic white	1.0	-
		
		Non-Hispanic black	1.11	0.63, 1.96
		
		Hispanic	2.03	1.07, 3.83
		
		Other	0.26	0.03, 1.91

Doctor visit (have you seen a doctor in the past year?) †	Indicator for diabetes knowledge and control	No	0.49	0.23, 1.05
		
		Yes	1.0	-

Insurance status (do you currently have health insurance?)	Indicator for diabetes knowledge and control	No	1.58	0.83, 3.02
		
		Yes	1.0	
		
		Missing	0.76	0.07, 8.42

BMI (kg/m^2^) ‡	Determinant of diabetes and indicator for selected lifestyle factors such as diet and physical activity	< 25	1.0	-
		
		25-29	1.85	0.93, 3.67
		
		≥ 30	4.29	2.25, 8.17

Smoking (do you now smoke cigarettes?)	Indicator for lifestyle factors	Yes ("everyday" or "some days")	1.0	-
		
		No ("not at all")	1.27	0.65, 2.49
		
		Missing	0.86	0.51, 1.45

* We combined Mexican Americans and other Hispanics in NHANES to match the race categories in BRFSS.

† This variable was defined as a composite of multiple questions about physician contact for specific conditions in the BRFSS.

‡ BMI was corrected for bias in self-reported height and weight in telephone interviews, using methods described elsewhere [2].

We estimated the individual-level probability of having diabetes in BRFSS 2003-2007 in two steps: First, participants who had answered "yes" to the diabetes diagnosis question were, by definition, assigned a probability of 1.0 for having diabetes. Second, the probability of having undiagnosed diabetes (i.e., FPG ≥ 126 mg/dL) for those who answered "no" to this question was estimated using the coefficients of the logistic regression fit on the NHANES dataset. Estimates of diabetes prevalence and diabetes diagnosis by age, sex, and state were obtained from the BRFSS using appropriate sample weights. The difference between total diabetes and self-reported diabetes is undiagnosed diabetes. In separate analyses, we used linear regressions to model the relationship between FPG as a continuous variable and self-reported diabetes diagnosis, medication use, and the health system, sociodemographic, and risk factor variables in Table 1 (results for continuous FPG analysis are available from authors by request). We used STATA version 10 for all analyses (StataCorp Texas). We present the results in two age groups: 30-59 and ≥ 60 years.

## Results

The national prevalence of diabetes among US adults ≥ 30 years was 13.7% (95% Confidence Interval 12.0%, 15.4%) for men and 11.7% (CI95 10.4%, 13.0%) for women in the pooled 2003-2006 NHANES. Nationally, approximately 32% of all diabetes cases in 2003-2006 were undiagnosed, a percentage that has changed little since 1999-2002 [4].

### Regression results

Among those who answered "no" to having been diagnosed with diabetes, being male and being older was associated with a higher probability of having diabetes (Table 1). The effect of age on diabetes risk was largest in those 60 to 69 years old and declined slightly in those ≥ 70 years old, consistent with the available evidence on the age association of blood glucose [8]. Overweight and obesity were associated with higher prevalence of undiagnosed diabetes, with obese participants (body mass index, BMI ≥ 30 kg/m^2^) having 4.29 times (95% CI 2.25, 8.17) the odds of having undiagnosed diabetes compared to normal weight. After controlling for all other factors, Hispanics had twice (95% CI 1.07, 3.83) the odds of having undiagnosed diabetes compared to whites, and the uninsured had 1.58 (95% CI 0.83, 3.02) times the odds compared to insured subjects.

We evaluated the performance of the prediction model using both internal and external validations. For internal validation, we applied the regression coefficients to NHANES 2003-2006 observations (i.e., the same data used in estimating the regression model) to predict diabetes prevalence. The differences between the predicted and actual diabetes prevalence for different age, sex, and race groups were on average 0.5 percentage points and at most 8.4 percentage points. The Pearson correlation coefficient for the observed and predicted diabetes prevalence for different age, sex, and race groups was 0.98. For external validation, we applied the coefficients of regressions estimated using the 2003- 2006 rounds to the same variables in pooled data from two previous rounds of NHANES (1999-2000 and 2001-2002). The observed-predicted differences for individual age, sex, and race groups were at the extreme slightly worse than those in the internal validation; specifically, the 60- to 69-year-old males from "other race" had a 20 percentage point discrepancy. This may, however, be because the composition of this race changed between the two surveys. The Pearson correlation coefficient for the observed and predicted diabetes prevalence for different age, sex, and race groups was 0.93. On average, the predicted prevalence was 0.1 percentage points higher than the actual prevalence (versus 0.5 lower percentage points in the internal validation).

### National-level prevalence of diabetes and undiagnosed diabetes

The predicted national prevalence of diabetes in 2003-2007 was 14.4% (14.3%, 14.5%) for men and 11.4% (11.3%, 11.5%) in women. The only sociodemographic group whose predicted and measured prevalences were significantly different was the uninsured, who had an actual prevalence of 9.2% (7.4%, 11.0%) but a predicted prevalence of 11.9% (11.6%, 12.2%).

### State-level prevalence of diabetes and undiagnosed diabetes

**Figure 1:**
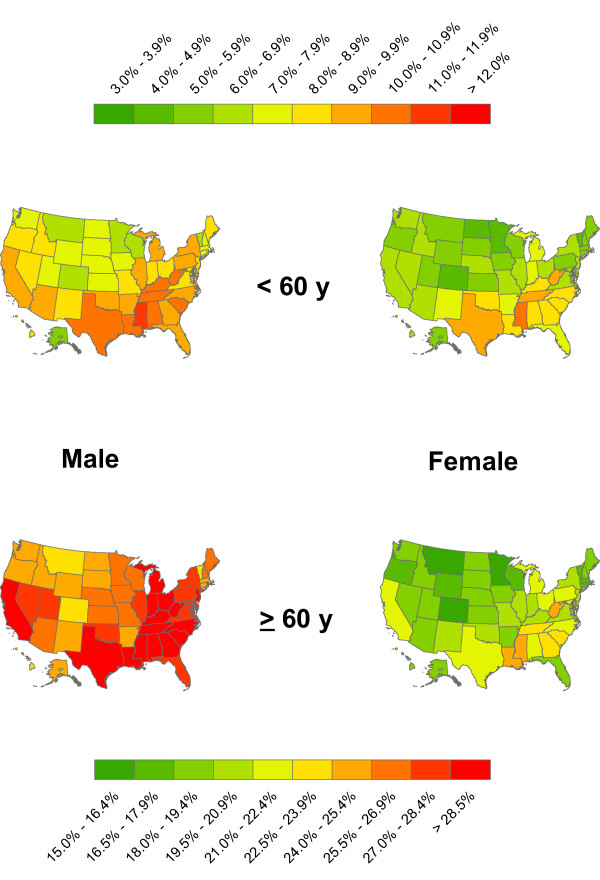
**Estimated prevalence of total diabetes by state, sex, and age group**. Within each age group, figures are age-standardized to the 2000 U.S. population.

In 2003-2007, the lowest prevalence of diabetes was in the Midwest and the Northeast, including Vermont, Minnesota, Montana, and Colorado, with age-standardized prevalence ranging from 11.0% to 12.2% for men and 7.3% to 8.4% for women (Figure 1 and Table 2). Diabetes prevalence was highest in the primarily Southern and Appalachian states, including Mississippi, West Virginia, Louisiana, Texas, South Carolina, Alabama, and Georgia, where age-standardized diabetes prevalence was 15.8% to 16.6% for men and 12.4% to 14.8% for women, i.e., approximately 30% to 51% higher for men and 48% to 103% higher for women than the states with lowest prevalence. The same geographic pattern was observed when younger (30-59 years) and older (≥ 60 years) age groups were considered separately. The Spearman rank correlation coefficient of state diabetes prevalence and mean BMI was 0.53 for men and 0.76 for women [2].

**Table 2:** Estimated prevalence (sampling standard error)* of total diabetes by state, age, sex, race and insurance status (Figures show actual prevalence; age-standardized figures available from authors).

State	Age group		Sex		Race				Insurance status	
	
	30-59 y	≥ **60 y**	Men	Women	White	Black	Hispanic	Other races	Insured	Uninsured
National NHANES	8.4% (.6)	23.6% (.1)	13.7% (.8)	11.7% (.7)	11.4% (.7)	18.3% (1.0)	16.7% (1.6)	11.1% (1.5)	13.3% (.7)	9.2% (.9)

National BRFSS Prediction	8.4% (.05)	23.8% (.1)	14.4% (.07)	11.4% (.05)	11.9% (.04)	17.3% (.18)	16.3% (.26)	11.3% (.29)	13.0% (.05)	11.9% (.15)

Alabama	10.1% (.3)	25% (.54)	16.1% (.45)	13.5% (.33)	13.6% (.3)	19% (.69)	14.5% (1.91)	11.7% (1.81)	15% (.3)	12.4% (.71)

Alaska	6% (.31)	21.8% (1.09)	9.9% (.5)	8.2% (.47)	9.2% (.38)	8.9% (1.17)	11.7% (2.25)	8.3% (1.01)	9.3% (.38)	7.8% (.77)

Arizona	8.4% (.41)	22.1% (.65)	14.5% (.58)	10.7% (.42)	11.7% (.38)	15% (1.66)	17.8% (1.28)	11.3% (1.32)	12.9% (.39)	11.1% (.94)

Arkansas	9.1% (.24)	21.4% (.42)	14.5% (.35)	11.7% (.27)	12.6% (.23)	17% (.8)	13.6% (1.66)	12.7% (1.33)	13.5% (.24)	10.5% (.51)

California	8.3% (.25)	25% (.59)	14.4% (.4)	11% (.3)	10.7% (.25)	15.4% (.86)	16.2% (.61)	10.5% (.94)	12.9% (.27)	11.2% (.72)

Colorado	5.7% (.17)	18.9% (.41)	10.6% (.26)	7.3% (.2)	8% (.17)	11.4% (.86)	15% (.67)	9.2% (1.13)	9.1% (.18)	7.7% (.45)

Connecticut	6.5% (.19)	20.6% (.4)	12.4% (.29)	9.3% (.23)	10.3% (.19)	16.7% (1)	14.6% (.97)	8.9% (1.22)	10.8% (.19)	10.8% (.68)

Delaware	8.3% (.31)	23.1% (.56)	15% (.45)	10.9% (.34)	12.4% (.3)	16.4% (.92)	12.5% (2)	9.3% (1.35)	13% (.29)	10.7% (.95)

District of Columbia	8.1% (.31)	26.3% (.78)	12.9% (.48)	13.3% (.44)	5.4% (.24)	18.1% (.52)	9.2% (1.24)	9.7% (1.72)	13.1% (.34)	12.8% (1.05)

Florida	9% (.25)	23.1% (.39)	16.2% (.36)	12% (.26)	13% (.23)	18% (.83)	15.9% (.7)	11.7% (1.54)	14.3% (.24)	12.3% (.61)

Georgia	9.2% (.25)	26.5% (.5)	14.6% (.37)	12.2% (.28)	12.2% (.24)	16.9% (.57)	12.2% (1.39)	9.9% (1.25)	13.5% (.25)	12.2% (.62)

Hawaii	7.6% (.3)	20.8% (.59)	12.9% (.44)	10.6% (.36)	8.7% (.34)	14% (.7)	14.2% (1.28)	12.4% (.46)	11.9% (.29)	9.3% (.89)

Idaho	7.7% (.24)	21.7% (.45)	12.8% (.35)	10.6% (.27)	11.4% (.23)	16.1% (1.97)	16.4% (1.45)	13.4% (1.45)	12% (.24)	10% (.56)

Illinois	8.6% (.27)	23.8% (.49)	14.5% (.4)	11.2% (.28)	11.2% (.22)	18.6% (.88)	17.6% (1.33)	9.8% (1.49)	12.5% (.24)	15.9% (1.06)

Indiana	8.4% (.22)	24.8% (.45)	14.7% (.33)	11.9% (.26)	12.9% (.22)	18% (1.01)	15.7% (1.7)	10.9% (1.35)	13.3% (.22)	12.3% (.63)

Iowa	6.9% (.2)	22.1% (.41)	13.4% (.31)	10.3% (.25)	11.8% (.2)	14.7% (1.69)	15.7% (1.85)	4.6% (1.15)	11.9% (.21)	10.5% (.69)

Kansas	7.3% (.17)	21.7% (.35)	13% (.26)	10.4% (.21)	11.3% (.17)	17.1% (.95)	14.3% (1.03)	10.6% (1.17)	11.8% (.17)	10.7% (.55)

Kentucky	9.8% (.27)	24.7% (.47)	15.7% (.4)	12.5% (.28)	13.7% (.24)	20.8% (1.55)	15.1% (1.96)	11.8% (1.83)	14.3% (.26)	12.2% (.61)

Louisiana	10% (.26)	26.6% (.5)	15.7% (.38)	13.6% (.29)	13% (.26)	18.8% (.56)	16.3% (1.61)	13.1% (1.31)	14.5% (.26)	15.1% (.59)

Maine	7.6% (.26)	22.2% (.52)	14.1% (.4)	10.3% (.29)	12.1% (.25)	16.6% (3.06)	11.2% (1.78)	11.4% (1.8)	12.4% (.26)	9.2% (.65)

Maryland	7.7% (.22)	24.7% (.51)	13.5% (.35)	11% (.28)	11.2% (.21)	15.4% (.57)	12% (1.71)	8.8% (1.12)	12.3% (.23)	10.6% (.77)

Massachusetts	6.4% (.16)	20.5% (.35)	12.2% (.25)	9% (.19)	10% (.16)	14.8% (.78)	17.6% (.91)	7.9% (.84)	10.5% (.16)	10.7% (.61)

Michigan	8.7% (.23)	25.1% (.43)	15% (.34)	12% (.26)	12.6% (.21)	18.2% (.81)	17.7% (1.83)	14.3% (1.55)	13.7% (.22)	10.6% (.65)

Minnesota	5.9% (.2)	20.1% (.45)	11.8% (.32)	8% (.23)	9.8% (.2)	11.9% (1.42)	12.2% (1.68)	10.4% (1.49)	10% (.21)	7.2% (.66)

Mississippi	11.4% (.27)	27.7% (.48)	16.9% (.39)	15.6% (.3)	14.3% (.27)	20.4% (.51)	16% (1.84)	16.9% (2.21)	16.6% (.27)	14.3% (.61)

Missouri	7.7% (.26)	22.9% (.52)	13.7% (.39)	11.3% (.31)	12% (.26)	16% (.98)	14.2% (2.4)	13.3% (1.65)	12.7% (.27)	10.4% (.62)

Montana	6.5% (.21)	19.3% (.43)	11.7% (.31)	9.3% (.26)	10.1% (.21)	19.3% (1.97)	13.4% (1.76)	14.3% (1.08)	10.6% (.22)	10% (.54)

Nebraska	7.3% (.21)	22.4% (.39)	13.3% (.31)	10.6% (.24)	11.7% (.2)	15.7% (1.53)	16.4% (1.39)	8.6% (1.36)	12% (.21)	11.1% (.61)

Nevada	7.5% (.34)	23.3% (.73)	13.9% (.5)	10.1% (.43)	12% (.38)	15.1% (1.4)	12.9% (1.03)	8.8% (.96)	12.2% (.35)	11.3% (.95)

New Hampshire	6.5% (.18)	22.2% (.43)	12.2% (.28)	9.5% (.23)	10.7% (.19)	12.1% (1.94)	17% (2.5)	12.2% (1.44)	11.1% (.2)	7.9% (.48)

New Jersey	7.9% (.18)	23.9% (.34)	14.4% (.27)	11% (.21)	11.3% (.16)	17.5% (.58)	16.1% (.69)	10% (.88)	12.7% (.17)	12.4% (.59)

New Mexico	8.4% (.22)	22.3% (.42)	13.5% (.31)	11.6% (.26)	10.2% (.23)	14.3% (.78)	16.4% (.43)	14.3% (1.15)	12.6% (.22)	12.3% (.49)

New York	8.5% (.23)	23.9% (.46)	14.7% (.35)	11.5% (.27)	11.5% (.21)	18.1% (.74)	15.6% (.86)	11.5% (1.08)	13.3% (.23)	10.9% (.69)

North Carolina	9.3% (.17)	25.6% (.33)	15.4% (.26)	12.8% (.2)	13% (.18)	19.2% (.44)	11% (.73)	12% (.92)	14.3% (.17)	12.3% (.41)

North Dakota	6.5% (.22)	21.7% (.49)	12.7% (.35)	10.1% (.3)	11.1% (.23)	14.9% (2.37)	19.1% (3.06)	15.5% (1.83)	11.2% (.23)	12.9% (.9)

Ohio	8.3% (.27)	24.6% (.57)	14.6% (.41)	11.9% (.33)	12.6% (.28)	18.4% (.96)	16.5% (2.91)	14.8% (2.05)	13.1% (.28)	13.9% (.94)

Oklahoma	9.7% (.22)	24.1% (.37)	15.7% (.31)	12.9% (.24)	13.1% (.2)	18.1% (.77)	18.3% (1.39)	17.4% (.8)	14.8% (.22)	11.4% (.47)

Oregon	7.1% (.21)	21.1% (.4)	13.1% (.32)	9.6% (.23)	11.2% (.2)	13.2% (1.14)	11.7% (1.34)	11.2% (1.36)	11.6% (.21)	9.0% (.56)

Pennsylvania	8.1% (.23)	24.1% (.43)	15.1% (.36)	11.6% (.25)	12.7% (.21)	17.7% (.99)	19.1% (2.16)	12.8% (1.71)	13.6% (.23)	10.4% (.66)

Rhode Island	7% (.23)	22.7% (.5)	13.7% (.37)	10.3% (.29)	11.7% (.24)	15.4% (1.39)	16.9% (1.31)	6.7% (1.15)	12.1% (.25)	10.3% (.78)

South Carolina	10% (.22)	26% (.4)	15.8% (.32)	13.4% (.26)	12.6% (.2)	20.4% (.55)	18.7% (1.91)	10.5% (1.17)	14.7% (.22)	14.0% (.57)

South Dakota	7% (.19)	21.5% (.37)	13% (.28)	10.3% (.23)	11.2% (.18)	17.7% (1.69)	16.5% (2.2)	18.9% (1.18)	11.7% (.19)	10.9% (.56)

Tennessee	10.5% (.37)	26.3% (.6)	16.3% (.53)	13.8% (.38)	14.6% (.32)	17.9% (1.18)	13.3% (2.41)	13.6% (2.67)	15.4% (.35)	12.0% (.78)

Texas	10.1% (.25)	25.4% (.46)	15.2% (.35)	13% (.28)	12.2% (.26)	17.1% (.65)	18.6% (.6)	12% (1.17)	14.3% (.25)	13.4% (.5)

Utah	6.3% (.22)	22.7% (.56)	12% (.35)	8.7% (.28)	10% (.23)	14% (1.96)	14% (1.4)	11.1% (1.59)	10.6% (.24)	8.1% (.62)

Vermont	6.1% (.16)	19.9% (.37	11.7% (.26)	8.6% (.2)	10% (.16)	13.3% (1.65)	14.9% (2.03)	11.8% (1.47)	10.3% (.17)	8.4% (.49)

Virginia	7.7% (.24)	23.6% (.58)	13.5% (.39)	10.5% (.31)	11.3% (.26)	16.8% (.81)	11.3% (1.15)	10% (1.49)	11.9% (.25)	12.6% (.95)

Washington	7.4% (.12)	21.3% (.23)	12.5% (.17)	9.9% (.13)	11.1% (.11)	13.5% (.69)	13.4% (.64)	10.7% (.56)	11.4% (.12)	10.0% (.35)

West Virginia	11.1% (.31)	27.3% (.53)	17.6% (.44)	15.4% (.36)	16.4% (.29)	19% (1.85)	15.1% (2.14)	17.1% (1.87)	17.2% (.31)	11.9% (.72)

Wisconsin	6.3% (.2)	21.6% (.49)	12.1% (.32)	9.4% (.26)	10.4% (.21)	17.2% (1.34)	14.6% (2.22)	10.6% (1.64)	10.7% (.22)	11.9% (.8)

Wyoming	7.3% (.22)	21.2% (.43)	12.8% (.32)	9.8% (.25)	10.8% (.2)	16.2% (2.07)	20.4% (1.58)	15.6% (1.86)	11.5% (.22)	10.0% (.54)

* The standard error of prevalence reported here reflects the sampling variability in the predicted diabetes prevalence but does not incorporate uncertainty in the prediction model (parameter uncertainty and stochastic uncertainty) and thus is an underestimate for the true standard error of prevalence. We included the parameter and stochastic uncertainty of the modeling using a multiple imputation approach in which we imputed the prevalence of diabetes for individuals who did not report having diabetes 10 times, drawing from a multivariate Normal distribution of the coefficients and drawing randomly from the posterior binomial distribution of revalence. We estimated the standard error of the national prevalence of diabetes using these 10 imputed values for each sex. The standard error was 0.8% for men and 0.4% for women, which is almost 10 times larger than the standard error estimated using sampling uncertainty only.

**Figure 2:**
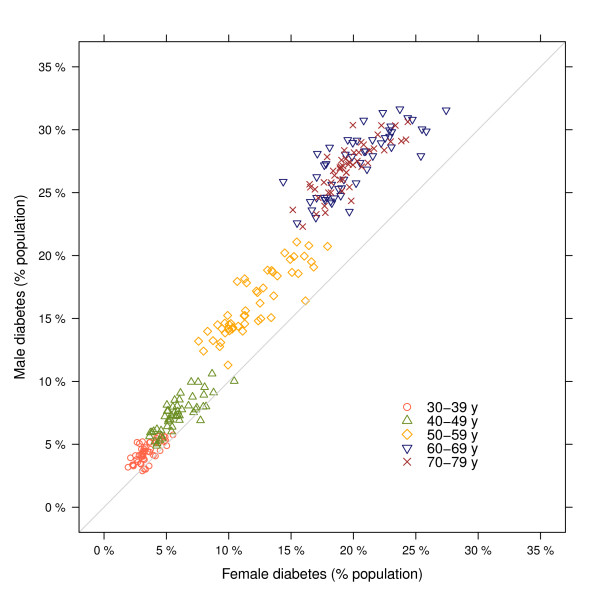
**Relationship between male and female diabetes prevalence, by age**. Each data point corresponds to one state.

Age-standardized diabetes prevalence was higher in men than women in all states, with the largest differences in Minnesota, Colorado, Utah, and Maine, where prevalence in men was 32% to 38% higher than among women. The smallest male-female differences were in the District of Columbia, Mississippi, West Virginia, and Louisiana, ranging from 6% to 18% (Figures 1 and 2). Men also had higher prevalence of diabetes than women in almost all states and age groups, except in the youngest ages (30 to 39 years), consistent with the national results from NHANES. Correlation between age-standardized male and female diabetes prevalence across states was 0.9.

The age-standardized proportions of diabetes cases that were undiagnosed were lowest in Hawaii, Mississippi, West Virginia, and Tennessee (19.5% to 21.4% of all diabetes cases) and highest in Minnesota, Montana, North Dakota, Vermont, and Colorado (31.1% to 33.3% of all diabetes cases). However, the absolute prevalence of undiagnosed diabetes, as a percent of total population, was highest in New Mexico, Texas, Florida, and California (3.5 to 3.7 percentage points) and lowest in Montana, Oklahoma, Oregon, Alaska, Vermont, Utah, Washington, and Hawaii (2.1 to 3.0 percentage points) (see Table 3 for prevalence of undiagnosed diabetes by age, sex, race and insurance status).

**Table 3:** Estimated prevalence (sampling standard error)* of undiagnosed diabetes by state, age, sex, race, and insurance (Figures show actual prevalence; age-standardized figures available from authors).

State	Age group		Sex		Race				Insurance status	
	
	30-59 y	≥ **60 y**	Men	Women	White	Black	Hispanic	Other races	Insured	Uninsured
National NHANES	2.1% (.4)	6.7% (.9)	4.5% (.6)	2.3% (.4)	3.4% (.5)	3.3% (.6)	4.4% (1.1)	.5% (.5)	3.4% (.4)	3.2% (.7)

National BRFSS Prediction	2.1% (<.01)	6.4% (.01)	4.3% (0.1)	2.5% (<.01)	3.3% (<.01)	3.3% (.02)	5.4% (.05)	.7% (.01)	3.3% (<.01)	3.9% (0.02)

Alabama	2.1% (.03)	6.2% (.09)	4.3% (.07)	2.5% (.03)	3.4% (.04)	3.5% (.08)	5.9% (.43)	.9% (.07)	3.3% (.04)	3.8% (.12)

Alaska	1.9% (.04)	6.3% (.18)	3.5% (.09)	1.9% (.05)	3% (.06)	3.5% (.21)	5.3% (.55)	.9% (.04)	2.7% (.06)	2.9% (.13)

Arizona	2.2% (.05)	6.3% (.12)	4.4% (.09)	2.5% (.05)	3.4% (.05)	2.8% (.15)	5.1% (.24)	.7% (.07)	3.4% (.06)	4% (.16)

Arkansas	2.1% (.02)	6.2% (.07)	4.4% (.06)	2.5% (.03)	3.5% (.03)	3.4% (.1)	5.6% (.39)	.9% (.04)	3.4% (.03)	3.7% (.1)

California	2.4% (.04)	6.3% (.11)	4.5% (.08)	2.5% (.04)	3.1% (.03)	3.4% (.12)	5.3% (.13)	.7% (.03)	3.3% (.04)	4.3% (.14)

Colorado	2% (.02)	6.2% (.07)	3.8% (.05)	2.2% (.02)	2.8% (.03)	2.9% (.11)	5.2% (.15)	.7% (.04)	2.9% (.03)	3.5% (.12)

Connecticut	2% (.02)	6.4% (.07)	4.2% (.05)	2.4% (.03)	3.3% (.03)	3.1% (.13)	5.1% (.22)	.6% (.04)	3.2% (.03)	4.1% (.15)

Delaware	2% (.03)	6.5% (.09)	4.3% (.08)	2.5% (.04)	3.5% (.05)	3.2% (.12)	4.7% (.44)	.7% (.05)	3.4% (.04)	3.5% (.18)

District of Columbia	1.9% (.03)	6.2% (.12)	3.8% (.08)	2.4% (.05)	2.4% (.04)	3.5% (.07)	4.4% (.3)	.6% (.05)	3% (.05)	4% (.2)

Florida	2.3% (.03)	6.9% (.08)	5.1% (.07)	2.9% (.04)	3.8% (.04)	3.5% (.11)	6.2% (.18)	.8% (.05)	3.9% (.04)	4.4% (.13)

Georgia	2% (.02)	6.1% (.08)	3.8% (.05)	2.2% (.02)	3.1% (.03)	3% (.06)	4.7% (.32)	.7% (.04)	2.9% (.03)	3.4% (.1)

Hawaii	1.4% (.02)	4.2% (.08)	3.1% (.06)	1.5% (.03)	3.4% (.06)	3.5% (.1)	4.9% (.23)	.9% (.02)	2.3% (.03)	2.7% (.14)

Idaho	1.9% (.02)	6.2% (.08)	4.1% (.06)	2.2% (.03)	3.2% (.03)	3.1% (.23)	5.2% (.31)	.7% (.04)	3.1% (.03)	3.4% (.1)

Illinois	2.1% (.03)	6.5% (.09)	4.2% (.07)	2.5% (.03)	3.3% (.03)	3.2% (.1)	5.4% (.25)	.6% (.04)	3.2% (.04)	4.0% (.15)

Indiana	2% (.02)	6.1% (.07)	4.1% (.05)	2.4% (.03)	3.3% (.03)	3.3% (.11)	4.8% (.27)	.8% (.05)	3.2% (.03)	3.5% (0.1)

Iowa	2% (.02)	6.6% (.07)	4.4% (.06)	2.6% (.03)	3.5% (.03)	3.5% (.23)	6% (.48)	.7% (.06)	3.5% (.03)	4.1% (.16)

Kansas	2% (.02)	6.2% (.06)	4.2% (.04)	2.4% (.02)	3.3% (.03)	3.2% (.11)	4.9% (.18)	.8% (.04)	3.3% (.02)	3.6% (.09)

Kentucky	2.1% (.03)	6.1% (.07)	4.2% (.06)	2.4% (.03)	3.3% (.03)	3.3% (.15)	5.8% (.44)	.8% (.07)	3.2% (.03)	3.4% (.09)

Louisiana	2.2% (.03)	6.7% (.09)	4.4% (.06)	2.6% (.03)	3.5% (.04)	3.5% (.07)	5.4% (.36)	1% (.06)	3.4% (.04)	3.8% (.09)

Maine	2% (.02)	6.3% (.09)	4.2% (.06)	2.4% (.03)	3.3% (.04)	3.1% (.42)	5.5% (.59)	.9% (.07)	3.3% (.04)	3.8% (.14)

Maryland	2% (.02)	6.4% (.08)	4.1% (.06)	2.3% (.03)	3.3% (.03)	3.3% (.07)	4.8% (.32)	.7% (.06)	3.1% (.03)	3.7% (.14)

Massachusetts	1.9% (.02)	6.3% (.06)	4.1% (.05)	2.3% (.02)	3.2% (.03)	3% (.13)	4.9% (.19)	.6% (.03)	3.1% (.03)	3.9% (.15)

Michigan	2% (.02)	6.4% (.07)	4.2% (.05)	2.4% (.03)	3.4% (.03)	3.2% (.09)	5.6% (.46)	.7% (.04)	3.3% (.03)	3.4% (.11)

Minnesota	1.9% (.02)	6.6% (.08)	4.1% (.06)	2.4% (.03)	3.3% (.03)	2.9% (.17)	6% (.51)	.6% (.05)	3.2% (.03)	3.6% (.15)

Mississippi	2.1% (.02)	6.1% (.08)	4.3% (.06)	2.4% (.03)	3.3% (.04)	3.3% (.06)	6.5% (.47)	.8% (.07)	3.3% (.03)	3.6% (0.1)

Missouri	2% (.03)	6.2% (.09)	4.2% (.07)	2.4% (.03)	3.4% (.04)	3.6% (.15)	4.8% (.37)	.8% (.06)	3.3% (.04)	3.5% (.13)

Montana	2% (.02)	6.3% (.08)	4.2% (.06)	2.4% (.03)	3.4% (.04)	3% (.19)	5.1% (.38)	.8% (.03)	3.3% (.04)	3.4% (.09)

Nebraska	2% (.02)	6.4% (.06)	4.3% (.05)	2.5% (.03)	3.4% (.03)	3.2% (.16)	5.3% (.28)	.8% (.06)	3.3% (.03)	3.9% (.11)

Nevada	2.1% (.04)	6.3% (.13)	4.3% (.09)	2.3% (.05)	3.4% (.06)	3.3% (.18)	5.4% (.26)	.7% (.04)	3.3% (.05)	3.8% (.19)

New Hampshire	1.9% (.02)	6.5% (.07)	4.1% (.05)	2.3% (.03)	3.2% (.03)	2.8% (.24)	5.3% (.54)	.8% (.06)	3.2% (.03)	3.2% (.11)

New Jersey	2.2% (.02)	6.6% (.07)	4.5% (.05)	2.6% (.02)	3.4% (.03)	3.5% (.08)	5.7% (.18)	.6% (.03)	3.4% (.03)	4.5% (.16)

New Mexico	2.6% (.03)	7% (.09)	4.9% (.07)	2.9% (.03)	3.3% (.04)	3.4% (.1)	5.7% (.1)	.8% (.03)	3.8% (.04)	4.4% (.12)

New York	2.1% (.03)	6.6% (.08)	4.4% (.06)	2.6% (.03)	3.4% (.03)	3.3% (.09)	5.8% (.21)	.8% (.04)	3.4% (.04)	4.1% (.16)

North Carolina	2.1% (.02)	6.2% (.05)	4.2% (.04)	2.4% (.02)	3.3% (.02)	3.6% (.06)	5.2% (.24)	.8% (.03)	3.2% (.02)	3.8% (.08)

North Dakota	2.1% (.03)	6.7% (.09)	4.5% (.07)	2.6% (.04)	3.6% (.04)	3.4% (.29)	7.6% (.73)	.9% (.07)	3.5% (.04)	3.8% (.15)

Ohio	2% (.03)	6.5% (.1)	4.3% (.07)	2.5% (.04)	3.4% (.04)	3.4% (.12)	6.3% (.68)	.8% (.06)	3.4% (.04)	3.6% (.14)

Oklahoma	2% (.02)	5.9% (.06)	4.1% (.05)	2.4% (.02)	3.4% (.03)	3.4% (.09)	5.8% (.31)	.9% (.03)	3.2% (.03)	3.3% (.08)

Oregon	1.9% (.02)	6.1% (.07)	4.1% (.05)	2.3% (.02)	3.3% (.03)	3% (.14)	4.1% (.23)	.8% (.04)	3.1% (.03)	3.4% (0.1)

Pennsylvania	2% (.02)	6.4% (.07)	4.3% (.06)	2.6% (.03)	3.5% (.03)	3.4% (.12)	5.1% (.38)	.7% (.06)	3.4% (.03)	3.7% (.14)

Rhode Island	2.1% (.03)	6.5% (.08)	4.4% (.07)	2.6% (.03)	3.5% (.04)	2.9% (.17)	5.2% (.26)	.8% (.06)	3.4% (.04)	4.3% (.17)

South Carolina	2.1% (.02)	6.1% (.06)	4.1% (.05)	2.4% (.02)	3.3% (.03)	3.3% (.06)	5.5% (.41)	.8% (.05)	3.2% (.03)	3.6% (.08)

South Dakota	2% (.02)	6.4% (.06)	4.4% (.05)	2.5% (.03)	3.5% (.03)	3.5% (.24)	6.4% (.5)	.8% (.03)	3.4% (.03)	3.7% (.12)

Tennessee	2.1% (.03)	6.1% (.09)	4.1% (.07)	2.4% (.03)	3.3% (.04)	3.2% (.12)	5.6% (.5)	.7% (.07)	3.2% (.04)	3.7% (.13)

Texas	2.5% (.03)	6.7% (.09)	4.5% (.06)	2.6% (.03)	3.3% (.03)	3.2% (.09)	5.4% (.11)	.7% (.04)	3.4% (.04)	4.3% (.09)

Utah	1.8% (.02)	6% (.09)	3.6% (.05)	2.1% (.03)	2.8% (.03)	2.8% (.19)	4.6% (.22)	.6% (.05)	2.8% (.03)	3.1% (.11)

Vermont	1.9% (.02)	6.3% (.06)	4% (.04)	2.3% (.02)	3.2% (.03)	3.7% (.26)	5.5% (.43)	.9% (.06)	3.1% (.03)	3.5% (.1)

Virginia	1.9% (.03)	6.2% (.09)	3.9% (.06)	2.3% (.03)	3.2% (.04)	3.2% (.09)	5.4% (.35)	.7% (.05)	3% (.04)	3.7% (.14)

Washington	1.9% (.01)	6.1% (.04)	3.9% (.03)	2.2% (.01)	3.1% (.02)	2.9% (.08)	4.6% (.13)	.7% (.02)	3% (.02)	3.3% (.06)

West Virginia	2.2% (.03)	6.2% (.08)	4.5% (.07)	2.6% (.03)	3.6% (.04)	3.8% (.23)	6.3% (.58)	.9% (.06)	3.6% (.04)	3.3% (0.1)

Wisconsin	1.9% (.02)	6.5% (.08)	4.2% (.06)	2.4% (.03)	3.3% (.04)	3.2% (.14)	4.9% (.4)	.8% (.07)	3.2% (.04)	4.0% (.15)

Wyoming	2.1% (.02)	6.5% (.08)	4.3% (.06)	2.4% (.03)	3.4% (.03)	3.5% (.24)	5.7% (.3)	.9% (.05)	3.3% (.03)	3.6% (.11)

* The standard error of prevalence reported here reflects the sampling variability in the predicted diabetes prevalence but does not incorporate uncertainty in the prediction model (parameter uncertainty and stochastic uncertainty) and thus is an underestimate for the true standard error of prevalence. See footnote to Table 2 for an example of how the inclusion of these sources would affect standard errors.

Men in all states had higher proportions of undiagnosed diabetes than women, with the male-female difference in undiagnosed proportion being largest in Hawaii, Mississippi, District of Columbia, West Virginia, and Idaho, where the proportion undiagnosed among men was 34.1% to 39.0% higher than among women. The male-female diagnosis disparity was smallest in Colorado, Pennsylvania, Vermont, and Minnesota (12.9% to 19.8%). When stratified on race, the proportion of cases undiagnosed was highest among Hispanics (33%), followed by whites (28%) and blacks (19%), and it was lowest in the residual group of "other races" (6%). One-third of diabetes cases were undiagnosed in participants who did not have insurance compared to one-fourth among insured Americans.

## Discussion

To our knowledge, this is the first study to estimate the total prevalence of diabetes and the proportion of diabetes that is undiagnosed at the state level. The Southern and Appalachian states had the highest diabetes prevalence, with Mississippi faring the worst. The Northern plains, the Northeast and the Midwest had the lowest prevalence. Prevalence of undiagnosed diabetes also varied across states, with Southern states and California having the highest prevalence. The proportion of undiagnosed diabetes was higher in men, Hispanics, and the uninsured compared to women, whites and insured. In fact, one-half of diabetes cases were undiagnosed in uninsured Hispanic men. These findings are important for the development and implementation of adequate state programs to prevent, diagnose, and control diabetes.

This analysis has a number of limitations: First, although our regression models included important sociodemographic, lifestyle, and health system determinants of diabetes risk and diagnosis, there are other factors that affect diabetes, such as diet and quality of care [9,10]. For instance, we were unable to include family history of diabetes, physical activity, alcohol use and specific dietary risk factors of diabetes [11-14] in the model because BRFSS does not include a sufficiently detailed dietary questionnaire or any questions on family history of diabetes and because the questions used to measure alcohol use and physical activity are different from those used in NHANES. The effects of some such factors may be captured by the variables in our model (e.g., self-reported diabetes, BMI, smoking, insurance status, visit to a doctor, etc.). If the unexplained effects vary systematically across states, the model may underestimate cross-state variation in diabetes prevalence, making our results conservative. Second, we conducted our analysis using FPG because of its availability for the most recent rounds of NHANES and because it is used by the American Diabetes Association to define diabetes. Other definitions of diabetes, e.g., based on glucose tolerance test, may have led to slightly different estimates. Third, BRFSS response rate varies across states. This may affect the state comparisons if the determinants of non-response are associated with diabetes prevalence. The single best way to reduce uncertainty in our analysis would be the addition of a validation component to BRFSS, which includes measured blood glucose for a random sample of interviewees. Finally, because 50.2% of observations in NHANES were missing either smoking or insurance status, we used a missing indicator in our regression models to include these observations. Dropping these observations would decrease the precision of our regression coefficients but would not affect the predictions of diabetes prevalence by states materially.

Despite uncertainties, our results currently provide the only estimates of total diabetes and undiagnosed diabetes in U.S. states, and should provide motivation, guidance, and benchmarks for designing, implementing, and evaluating diabetes prevention and control programs at the state level. Further, our methods allow states to combine the relatively low-cost BRFSS telephone survey with NHANES to regularly monitor the prevalence of diabetes and progress in diabetes diagnosis.

Increasing the coverage of lifestyle, e.g., physical activity and pharmacological interventions for diabetes, should be a priority in states with high diabetes prevalence. Some states also need to improve diagnosis, especially among men, because early diagnosis and intensive glycemic control reduces the future incidence of microvascular complications [15,16]. Further, diabetes diagnosis will facilitate interventions that lower blood pressure and cholesterol, and hence the risk of cardiovascular disease, among diabetics [17,18]. The states with the highest estimated diabetes prevalence in our analysis also have the highest levels of blood pressure and cardiovascular disease risk [19,20]. This geographical distribution of cardiovascular risks and diabetes points to the need for lifestyle and health care interventions that reduce blood pressure and other cardiovascular risks in high-diabetes states.

## Competing interests

The authors declare that they have no competing interests.

## Authors' contributions

GD, CJM and ME designed the study. GD, ABF and SO conducted the analyses. GD and ME wrote the paper with input from other authors. ME oversaw the research and acts as the paper's guarantor. All authors have read and confirmed the final manuscript.
